# A51 ROLE OF GUT MICROBIOTA IN THE EPISODIC NATURE OF SYMPTOMS IN IRRITABLE BOWEL SYNDROME

**DOI:** 10.1093/jcag/gwab049.050

**Published:** 2022-02-21

**Authors:** V Mohan, M I Pinto-Sanchez, A Nardelli, R Borojevic, G De Palma, S M Collins, P Bercik

**Affiliations:** 1 McMaster University Faculty of Health Sciences, Hamilton, ON, Canada; 2 Medicine, McMaster University, Hamilton, ON, Canada; 3 McMaster University, Hamilton, ON, Canada

## Abstract

**Background:**

Irritable bowel syndrome (IBS) is a complex functional gastrointestinal disorder with likely heterogenous pathophysiology, multiple symptoms, and comorbidities. Growing evidence shows that the gut microbiota composition and function are altered in IBS patients. However, identifying the critical drivers of clinical expression remains challenging due to the episodic occurrence of IBS symptoms, the inherent variability in composition of gut microbiota across individuals, and high sensitivity of gut microbiota to dietary and environmental cues.

**Aims:**

To identify whether changes in gut microbiota composition accompany or, predict the occurrence of symptoms.

**Methods:**

28 IBS patients (IBS-D n=20, IBS-C n=8) and 10 healthy controls (HC) were followed longitudinally for 25 weeks, collecting stool samples, and recording their symptoms weekly. Stool microbiota profiles were assessed by 16S rRNA gene sequencing using Illumina platform. The sequences were preprocessed, filtered, and annotated using DADA2 and phyloseq pipelines; statistical analyses were performed using FactomineR and microbiomeanalyst packages in R. Statistical significance was set at p<0.05.

**Results:**

Multifactorial analysis of clinical data classified 950 samples in 6 clusters. Distribution of samples among the clusters was based on Bristol stool scale defining symptomatic periods (scores <3 and >4 indicating abnormal stool) and asymptomatic periods (scores 3 or 4), with several gut and mood symptoms varying significantly between the two categories. IBS-D patients, but not IBS-C patients presented with changes in symptoms severity, such as pain, diarrhea, constipation, and anxiety during the symptomatic periods. Depression scores were, however, higher in IBS-C compared to IBS-D patients. In contrast, immune makers such as fecal b-defensin-2 and calprotectin were higher during asymptomatic periods in IBS-D, but not in IBS-C patients. Bacterial diversity profiles differed among IBS patients (IBS-D and IBS-C) and HC, namely Shannon index and Bray-Curtis distance, but they did not change significantly between the symptomatic and asymptomatic periods within each subtype. Despite this, several bacterial taxa unique to each cluster were identified using linear mixed models.

**Conclusions:**

Our results demonstrate the need to study patterns of co-occurrence of IBS symptoms and their severity during symptomatic and asymptomatic periods to better understand the role of identified bacterial taxa in the symptom generation. Identifying their temporal changes and cross-feeding patterns in individual patients will shed light on the underlying mechanistic role of gut microbiota in IBS, which might be otherwise obscured by group generalizations.

**Funding Agencies:**

CIHR

